# Why bees are critical for achieving sustainable development

**DOI:** 10.1007/s13280-020-01333-9

**Published:** 2020-04-20

**Authors:** Vidushi Patel, Natasha Pauli, Eloise Biggs, Liz Barbour, Bryan Boruff

**Affiliations:** 1 UWA School of Agriculture and Environment, The University of Western Australia (M004), 35 Stirling Highway, Crawley, WA 6009 Australia; 2 Cooperative Research Centre for Honey Bee Products, 128, Yanchep Beach Rd, Yanchep, WA 6035 Australia; 3 Department of Geography and Planning, The University of Western Australia (M004), 35 Stirling Highway, Crawley, WA 6009 Australia

**Keywords:** Bees, Biodiversity, Complex systems, Human–environment interactions, Pollination, Sustainable Development Goals

## Abstract

Reductions in global bee populations are threatening the pollination benefits to both the planet and people. Whilst the contribution of bee pollination in promoting sustainable development goals through food security and biodiversity is widely acknowledged, a range of other benefits provided by bees has yet to be fully recognised. We explore the contributions of bees towards achieving the United Nation’s Sustainable Development Goals (SDGs). Our insights suggest that bees potentially contribute towards 15 of the 17 SDGs and a minimum of 30 SDG targets. We identify common themes in which bees play an essential role, and suggest that improved understanding of bee contributions to sustainable development is crucial for ensuring viable bee systems.

## Introduction

The United Nations’ 17 Sustainable Development Goals (SDGs) are designed to achieve synergy between human well-being and the maintenance of environmental resources by 2030, through the pursuit of 169 targets and more than 200 indicators (UN 2015). The biosphere is the foundation for all SDGs (Folke et al. 2016; Rockström and Sukhdev 2016; Leal Filho et al. 2018), and yet biodiversity conservation remains a persistent global challenge (Tittensor et al. 2014). An examination of how a particular suite of organisms within the global wealth of biodiversity can contribute to the attainment of the SDGs holds the potential to link sustainable development policy with conservation through the design of integrated solutions. We explore the interconnections between bees—a critical group of insects with diverse economic, social, cultural and ecological values—and people, in the context of the SDGs.

## Bees, people and the planet

**Fig. 1:**
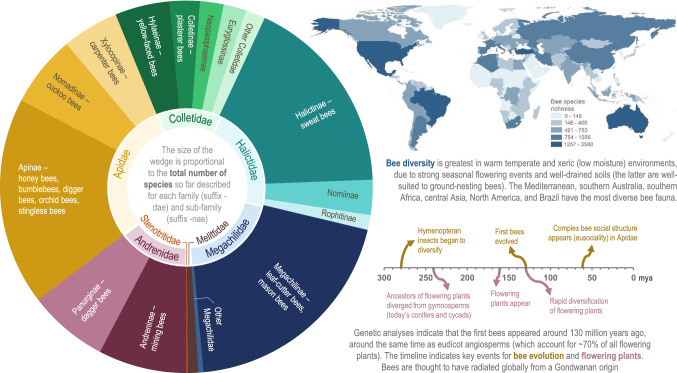
A snapshot of the diversity of bees. Bees are taxonomically classified under the insect Order Hymenoptera, along with ants, wasps and sawflies, and are part of the superfamily Apoidea, and clade Anthophila, with seven recognised families. Although only 50 of the ~ 20 000 described bee species are actively managed by people, the entire clade is important for ecosystem functioning and human well-being. Bees and flowering plants have co-evolved, making bees effective pollinators of a large proportion of flowering plant species. There are perhaps a further ~ 5 000 bee species that are yet to be described. Data source: Ascher and Pickering (2014). Information for this figure was sourced from Michener 1979; Michener 2000; Michez and Patiny 2007; Litman et al. 2011; Cappellari et al. 2013; Peters et al. 2017; Meiners et al. 2019

Bees comprise ~ 20 000 described species across seven recognised families (Ascher and Pickering 2014), with many more species yet to be described (Fig. 1). The evolutionary radiation of bees coincided with the evolutionary radiation of flowering plants (Cappellari et al. 2013), and bees occupy an important ecological role as pollinators of a range of flowering plant species. Although bees are not the most diverse group of pollinators (butterflies and moths comprise over 140 000 species), they are the most dominant taxonomic group amongst pollinators; only in the Arctic regions, is another group (flies) more dominant (Ollerton et al. 2017). The ability of bees to transport large numbers of pollen grains on their hairy bodies, reliance on floral resources, and the semi-social or eu-social nature of some species are amongst the characteristics that make bees important and effective pollinators (Ollerton et al. 2017; Klein et al. 2018). Fifty bee species are managed by people, of which around 12 are managed for crop pollination (Potts et al. 2016a).

The potential importance of bees for crop pollination has been highlighted as a particular reason to conserve wild bees and their habitat (Klein et al. 2007; Gill et al. 2016; Potts et al. 2016a; Klein et al. 2018). More than 90% of the world’s top 107 crops are visited by bees; however, wind- and self-pollinated grasses account for around 60% of global food production and do not require animal pollination (Klein et al. 2007). Wild bees contribute an average of USD$3 251 ha^−1^ to the production of insect-pollinated crops, similar to that provided by managed honey bees (Kleijn et al. 2015). A very small number of mostly common wild bee species provide the majority of bee-related crop pollination services (Kleijn et al. 2015), and other insects such as flies, wasps, beetles, and butterflies have an important, underemphasised role in crop pollination (Rader et al. 2016). Such research has highlighted the danger of exclusively highlighting the importance of bees for crop pollination, to the potential detriment of conserving diversity across the landscape (Kleijn et al. 2015; Senapathi et al. 2015). In our assessment of bees and the SDGs, we highlight that the diversity of wild and managed bees has crucial ecological, economic and social importance including and beyond crop pollination.

Long-standing associations exist across multiple bee species and human societies. Documented ancient bee–people interactions include honey hunting dating back to the Stone Age for the honey bee *Apis mellifera* in Europe (Roffet-Salque et al. 2015), more than 2 000 years of keeping the honey bee *Apis cerana* in Asia (Crane 1995), and beekeeping reaching back to at least pre-Columbian times for stingless bees (*Melipona beechii*) in Mayan Mexico (Quezada-Euán 2018). Bees also appear in many religious scriptures and are found within mythology, cosmology and iconography (Fijn 2014; Roffet-Salque et al. 2015; Potts et al. 2016a; Quezada-Euán 2018). Beeswax from culturally significant sugarbag bees (*Tetragonula* spp.) has been used in the production of rock art by Aboriginal peoples in northern Australia for at least 4 000 years (Watchman and Jones 2002). In Greek society, bees are closely linked with the cycle of birth and death, and considered an emblem of immortality (Cook 2013). “Telling the bees” was a popular tradition in 19th Century New England; it was customary for keepers to inform their bees of any major event such as a birth, death, marriage or long journey (Hagge 1957). These reciprocal bee–human relationships have historic legacy and are highly important for informing current practices around bee management.

Today, the long-standing mutualistic relationship between bees and people is jeopardised by recent reported declines in bee populations (Potts et al. 2016b). The loss of managed honey bee colonies (e.g. Potts et al. 2010) and declines in wild bee pollinators (e.g. Biesmeijer et al. 2006; Koh et al. 2016) have been observed, particularly in Europe and North America. However, much remains undocumented about the conservation status of most bee species (Goulson et al. 2015; Jamieson et al. 2019). The global conservation status of just 483 bee species has been assessed by the IUCN, most of which were ‘data deficient’ (IUCN 2019). The European Red List assessment of 1 965 species of European bees found that 9.2% were threatened, whilst insufficient data were available to assess the conservation status of nearly 57% of European species; many of these may also be threatened (Nieto et al. 2014). Goulson et al. (2015) reason that declines in wild bees definitively noted for Europe and North America are likely to have occurred elsewhere.

With a decline in bee populations, there has been a surge of research focusing on the drivers of bee decline and the impacts on provisioning ecosystem services (Goulson et al. 2015; Decourtye et al. 2019). Drivers such as habitat loss, pesticide use, the proliferation of parasites, availability and diversity of forage, change in land use and climate, and species competition have all contributed to the reduction in bee populations (Goulson et al. 2015; Sánchez-Bayo and Wyckhuys 2019; Wagner 2020). These drivers interact in complex ways; for example, market-driven agricultural intensification has limited bees’ access to forage resources and at the same time potentially increasing bees’ exposure to harmful agrichemicals (Durant 2019; Sánchez-Bayo and Goka 2014). People can act as a positive influence for ecosystem function through designing bee-friendly policies and contributing to bee conservation approaches (Potts et al. 2016a; Matias et al. 2017; Hill et al. 2019). Acknowledging the plethora of literature addressing the decline in bee populations and the consequences for agriculture, we contend that the ubiquitous importance of bees in connecting the planet and people remains relatively less explored, particularly with regard to broader goals in sustainable development.

## Framing the broader importance of bees to sustainable development

Bees provide a range of ecosystem services that contribute to the wellbeing of people whilst maintaining the planet’s life support systems (Gill et al. 2016; Matias et al. 2017). Ecosystem services inherently contribute to achieving global sustainable development (Wood et al. 2018). Yet the extent to which bees contribute towards the achievement of the full suite of the SDGs has not been explored in detail. Existing research has highlighted the importance of insects in achieving multiple SDGs through the regulation of natural cycles, biological pest control, pollination, seed dispersal, and even as bio-inspiration (Gill et al. 2016; Sánchez-Bayo and Wyckhuys 2019; Dangles and Casas 2019). Bee pollination has been identified as directly contributing to food security (SDG2) and biodiversity (SDG15) (Dangles and Casas 2019). However, bees could also contribute to a broader range of SDGs.

We explicitly identify the realised and potential contributions of bees towards achieving the SDGs, presenting evidence to highlight the interconnectedness between bees, people and the planet from an integrated system perspective (Stafford-Smith et al. 2017). We review the SDGs alongside the potential contributions of bees in achieving individual SDG targets. As the SDGs explicitly build on the foundation of the biosphere (Folke et al. 2016; Leal Filho et al. 2018), the perspective presented here may help in designing implementation pathways to achieve SDG targets. We identify 30 targets to which bees may contribute (Table 1) through a range of direct and indirect connections between bees, people and the planet.

**Table 1:** The contributions of bees towards relevant SDG targets

Sustainable development goal (SDG)^a^	Contributions from bees to SDG targets	Examples of supporting literature^b^	Details on the contributions that bees may provide towards achieving the SDG targets
1. No Poverty	1.1 1.4 1.5	Bradbear, 2009; Amulen et al. 2019; Pocol and McDonough 2015	Keeping bees offers economic diversity as an income source (1.1) helping build resilient livelihoods for poor and vulnerable peoples (1.5), whilst potentially providing equal access to economic and natural resources for both men and women (1.4)
2. Zero hunger	2.2 2.3	Klein et al. 2007; Kleijn et al. 2015; Potts et al. 2016a; Stein et al. 2017; Klein et al. 2018	Bee pollination increases crop yield (2.3) and enhances the nutritional value of fruits, vegetables, and seeds (2.2)
3. Good health and well-being	3.4 3.8 3.9	Bradbear, 2009; Brockerhoff et al. 2017; Pasupuleti et al. 2017; Sforcin et al.^2017^; Kocot et al. 2018; Easton-Calabria et al. 2019;	Bee products provide safe and affordable medicinal sources (3.8) used in traditional and modern medicine to treat non-communicable diseases such as cancer through strong bioactive compounds (3.4). Bee pollination potentially contributes to the growth and diversity of plants that are important for improved air quality (3.9)
4. Quality education	4.3 4.4 4.5	Pocol and McDonough 2015; Mburu et al. 2017; Ekele et al. 2019	Vocational training for keeping bees can enhance equal opportunities for employment, training and entrepreneurship amongst men, women and indigenous people (with traditional knowledge) (4.3, 4.4 and 4.5).
5. Gender equality	5.5 5.a	Pocol and McDonough 2015; Mburu et al. 2017	Keeping bees as a hobby or being involved in beekeeping can enhance opportunities for women’s involvement in economic, social and political decision-making processes even in communities that deprive women of property rights (5.5, 5.a)
6. Clean water and sanitation	6.6	Brockerhoff et al. 2017; Creed and van Noorwijk 2018	Bee pollination may contribute to growth and diversity in water-related ecosystems, such as mountains and forest. Appropriate afforestation efforts may provide new resources for commercial bee operations whilst potentially contributing to regional water supply (6.6)
7. Affordable and clean energy	7.2	Romero and Quezada-Euán 2013; Halinski et al. 2018; Perrot et al. 2018	Bee pollination improves production for oilseed crops used as biofuel such as sunflower, canola and rapeseed (7.2)
8. Decent work and economic growth	8.1 8.6 8.9	Arih and Korošec 2015; Mazorodze 2015; Pocol and McDonough 2015; Stein et al. 2017; Quezada-Euán 2018; Vinci et al. 2018	Improved agricultural production from bee pollination may contribute to the gross domestic product (GDP) of nations (8.1). Beekeeping can diversify livelihood opportunities for men and women in rural areas (8.6) and support nature-based tourism initiatives (8.9).
9. Industry innovation and infrastructure	9.b	Xing and Gao 2014; Zhang et al. 2015; Sahlabadi and Hutapea 2018	Bees are an element of nature that inspires human innovations (e.g., airplane design and computer algorithm development) and new honey-related products (9.b)
10. Reduced inequality	10.1 10.2	Carroll and Kinsella 2013; Tomaselli et al. 2014; Mburu et al. 2017	Improved livelihoods from beekeeping and the contribution of bee pollination towards GDP can support sustainable income growth for lower income groups (10.1) which can potentially contribute to promoting inclusive social, economic and institutional development (10.2)
11. Sustainable cities and communities	11.6 11.7	Lowenstein et al. 2015; Van der Steen et al. 2015; Hausmann et al. 2016; Stange et al. 2018; Zhou et al. 2018	Bees can be useful in monitoring air quality in urban areas, as pollination of urban flora can support improved local air quality (11.6). Bees can enhance pollination and self-sustainability of urban gardens and public open spaces (11.7)
12. Responsible consumption and production	12.3 12.b	Klatt et al. 2014; Lemelin 2019	Bee pollination can contribute to reducing food waste by improving visual aesthetics of food (shape, size and colour) and increase shelf life (12.3). Beekeeping can be marketed as sustainable tourism for regional development (12.b)
13. Climate actions	13.3	Van der Steen et al. 2015; Smith et al. 2019	Use of bees and bee products for environmental monitoring can improve understanding of climate impacts on the environment (13.3)
14. Life below water	14.4	Amjad Khan et al. 2017	Bees can potentially contribute to improved production of plant-based sources of compounds commonly found in fish. Overharvesting of fish can be managed by promoting production and consumption of alternative plant-based nutrient sources (14.4)
15. Life on land	15.1 15.5 15.9	Senapathi et al. 2015; Minja and Nkumilwa 2016; Chanthayod et al. 2017; Klein et al. 2018; Mudzengi et al. 2019	Bees contribute to biodiversity by pollinating flowering trees and plants (15.5) and beekeeping can contribute to forest conservation (15.1). Incorporating beekeeping in local planning processes may support reforestation activities which can result in poverty reduction and sustainable regional development (15.9).

^a^SDG16 (peace, justice and strong institutions) and SDG17 (partnership for the goals) were excluded from this analysis given their focus on governance and policy

^b^Supporting literature includes a mix of direct and indirect evidence. The details on bees’ potential contribution to SDGs have been provided using the language used in SDG targets, which may differ from the language used in the supporting literature

We incorporate contributions from all bee species, including wild and managed populations. The European honey bee (*A. mellifera*) and buff-tailed bumblebee (*Bombus terrestris*) could be considered as ‘massively introduced species’ having greatly expanded their geographic range through human management and escape (Geslin et al. 2017). We note the extensive and evolving literature on the interactions between native wild bees, introduced domesticated bees, and feral bees, noting evidence of competition for forage and nesting resources, disruption of native plant–pollinator networks, and potential for viral disease transmission between species (e.g. Geslin et al. 2017; Mallinger et al. 2017; Wojcik et al. 2018; Alger et al. 2019; Murray et al. 2019; Valido et al. 2019). We pursue a holistic perspective that encompasses native wild and managed introduced bees, following Kleijn et al.’s (2015, 2018) calls for an inclusive approach that safeguards all pollinators.

## The identified critical role of bees in sustainable development

The importance of bee pollination for food crops has been widely acknowledged, with growing concern of a global crisis as demand for pollination services continues to outstrip supply, with an associated increase in less diverse, pollinator-dependant agriculture systems (Aizen and Harder 2009; Aizen et al. 2019). In addition to improving the yield of some crops (target 2.3) (Klein et al. 2007, 2018; Stein et al. 2017), bee pollination contributes to enhanced nutritional value (target 2.2) and improved quality and longer shelf life of many fruits and vegetables (Klatt et al. 2014), which could potentially help in reducing food waste (target 12.3) resulting from aesthetic imperfections (Gunders and Bloom 2017).

Less-explored aspects of bee pollination include the contribution to biofuels (SDG7). Despite being self-pollinated, oil seed crops show increased yield when pollinated by bees (target 7.2) (Halinski et al. 2018; Perrot et al. 2018). Research in Mexico on the performance of bees on *Jatropha curcas* found significant improvement in the seed set when the self-pollinated varieties were supported with bee pollination (Romero and Quezada-Euán 2013). Canola, another self-pollinating oilseed crop, also shows a positive association between higher yields and bee diversity (Halinski et al. 2018).

Beyond agricultural landscapes, research in urban bee ecology aids understanding of bee dynamics in our cities and informs urban bee conservation initiatives (Hernandez et al. 2009; Stange et al. 2017). Urban beekeeping strengthens residents’ connection to nature (Stange et al. 2018). Planting aesthetically pleasing, bee-attractive flowering species in landscape planning can provide forage for bees, and close proximity to such plantings may result in pollination rewards for trees and other species in public green spaces (target 11.7) (Lowenstein et al. 2015; Hausmann et al. 2016). European honey bees can be used as an indicator species for tracking contaminants and monitoring environmental health (target 13.3) in urban areas (Zhou et al. 2018). In addition, understanding bee forage preference, suitability of habitat and mobility between different habitat types is critical for designing sustainable urban (target 11.7) and rural landscapes (target 15.9) to optimize pollination benefits as well as support bee health (Stange et al. 2017; Langellotto et al. 2018). For example, the United Kingdom’s Protection of Pollinators Bill was proposed to develop a national network of wildflower corridors called B-lines to support bee populations and other pollinators (UK Parliament, House of Commons, 2017).

The contribution of wild and managed bees in pollinating wild plants in natural ecosystems and managed forests (target 15.1) is well-acknowledged (Senapathi et al. 2015; Klein et al. 2018). The biodiversity found within forests provides a critical range of ecosystem services including water cycle regulation (target 6.6) and carbon sequestration (Brockerhoff et al. 2017; Creed and van Noordwijk 2018). Bee-pollinated plants provide a source of food for wildlife and non-timber forest products for people (Bradbear, 2009; Senapathi et al. 2015). For example, Brazil nut trees (*Bertholletia excelsa*) require bee pollination to set their high-value fruit, with much greater productivity in the wild, likely due to low numbers of native bees in plantations (Cavalcante et al. 2012). Beekeeping within forest boundaries can support forest conservation (target 15.1) alongside rural livelihoods (Sande et al. 2009; Chanthayod et al. 2017; Mudzengi et al. 2019).

Keeping bees provides opportunities for income diversity (target 1.1) with low start-up costs, through diverse products and services including honey, pollen, beeswax, propolis, royal jelly, and pollination services (Bradbear 2009). Initiatives to promote beekeeping and pollination services in Kenya have resulted in livelihood improvements for smallholder farmers through increased farm productivity and an additional income stream (target 1.5) (Carroll and Kinsella 2013). However, in other regions of Africa, constraints to improve livelihoods through bee-related activities have been attributed to a lack of knowledge concerning bee husbandry processes, access to equipment, and training (Minja and Nkumilwa 2016). Vocational education in beekeeping (target 4.3) could promote economic opportunities for employment and entrepreneurial enterprise (targets 8.6 and 4.4) and diversification for Indigenous groups (targets 1.4 and 4.5), as well as help empower women (target 5.5) including those within traditionally patriarchal societies to promote gender equality (target 5.a) (Pocol and McDonough 2015; Mburu et al. 2017).

Beekeeping can be an important strategy for livelihood diversification (Bradbear 2009), which can directly contribute to an increase in per capita and household income (target 8.1) (Mazorodze 2015; Chanthayod et al. 2015) and also allow for enhanced fiscal opportunities (e.g. tourism) and sustained income growth for people in rural areas, irrespective of social and economic status (targets 10.1 and 10.2) (Pocol and McDonough 2015; Vinci et al. 2018). An initiative for sustainable tourism in Slovenia packages bee-related education and healing experiences with bee products, together with opportunities to create and purchase original crafts using bee products (Arih and Korošec 2015). In Fiji, The Earth Care Agency is working to promote organic honey production on remote islands to provide economic alternatives for indigenous Fijians (Matava Fiji Untouched 2019). These initiatives contribute to local economies and in the case of Slovenia (Arih and Korošec 2015), help in marketing the country’s natural attractions whilst providing additional livelihood opportunities through increased tourism activities (target 8.9).

In relation to health, honey, bee pollen, propolis, royal jelly, beeswax and bee venom have all been used in traditional and modern medicine (target 3.8) (Kocot et al. 2018; Easton-Calabria et al. 2019). Researchers have identified bioactive properties of honey, propolis and royal jelly which suggest the presence of compounds with antimicrobial, anti-inflammatory, antioxidant, antitumor, and anticancer activities (Pasupuleti et al. 2017; Kocot et al. 2018; Easton-Calabria et al. 2019). Honey is used in wound and ulcer care, to enhance oral health, fight gastric disorders, and liver and pancreatic diseases, as well as to promote cardiovascular health (Pasupuleti et al. 2017; Easton-Calabria et al. 2019). Propolis is used in gynaecological care, oral health, dermatology care, and oncology treatments, whilst royal jelly is used in reproductive care, neurodegenerative and aging diseases, and wound healing (target 3.4) (Pasupuleti et al. 2017).

Bees have contributed to industry, innovation and infrastructure by inspiring the design and development of a range of structures, devices and algorithms that can benefit sustainable development (target 9b). The honeycomb structure of beehives is often a mainstay in structural engineering (Zhang et al. 2015). Drawing inspiration from bee anatomy, the medical industry has benefited from innovations such as surgical needles adopted from the design of bee stingers (Sahlabadi and Hutapea 2018). Bee behaviour has inspired complex computer-based search and optimisation processes informing a new wave of genetic algorithms (Xing and Gao 2014).

## Towards sustainable bee systems

The decline in global insect populations has attracted the attention of the scientific community, general public and policymakers (Potts et al. 2016a), with heightened public awareness of the importance of bees for pollination. Our research has highlighted the contribution bees can provide towards achieving a diverse range of SDG targets in addition to their crucial role in pollination. The increasingly positive attitude of the public towards bees, and insect pollinators more broadly, provides opportunities for efforts to conserve bee habitat and support pro-pollinator initiatives in land management, agricultural diversification and urban greening (Senapathi et al. 2015; Schönfelder and Bogner 2017).

**Fig. 2:**
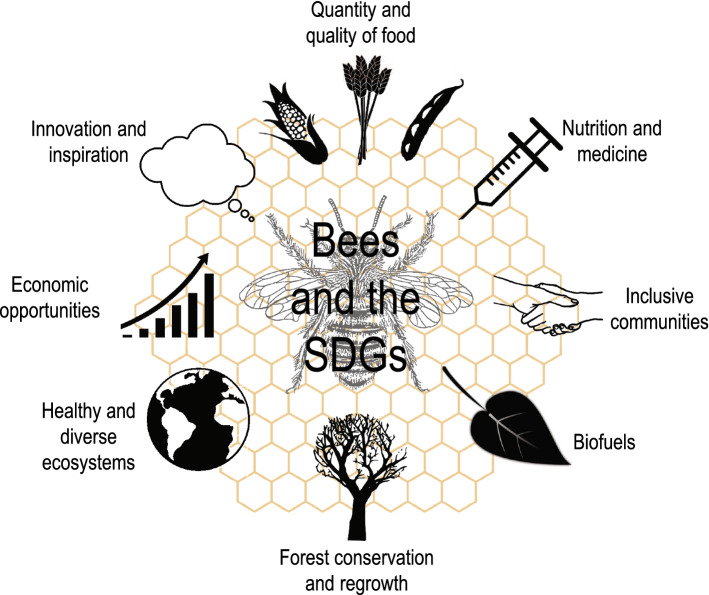
Bees and the SDGs. Overarching themes whereby bees contribute to sustainable development targets

A holistic view of ecosystems including wild and managed bees and humans is necessary to address sustainability challenges (Kleijn et al. 2018; Saunders et al. 2018). By employing a system approach, we can better understand the interconnections between elements within coupled human–environment systems. We strongly advocate the need for appropriate natural resource management approaches for maintaining sustainable systems as vital for allowing the continued success of bees in their natural role. We summarise our findings by suggesting eight key thematic priority areas whereby bees can play a crucial role in meeting the SDGs (Fig. 2).

These themes provide a foundation for an emerging, yet urgently needed research agenda to explore the complex relationship between bees, people and the planet. A range of important questions should guide this research agenda including: (i) What social and ecological entities contribute to a bee–human system, what feedback and trade-offs exist amongst these entities, and how can understanding structural interconnectivities within this bee–human system contribute to sustainable decision making at various spatial scales? (ii) Are there critical thresholds of bee species diversity and/or bee population abundance beyond which there are significant impacts to meeting certain SDG targets, and do these thresholds vary by geographic regions? (iii) What ecosystem services can be optimized with existing bee diversity in a region, to what extent can they contribute to achieving SDG targets, and does the introduction of managed species enhance or suppress existing ecosystem services? In addition, the distinct roles of wild and managed bees provide a further research lens for identifying the critical role that bees can provide in achieving the SDGs. We must strive to restore balance and reverse bee decline trajectories if we are to encounter a future in which bees continue to contribute to the sustainable development of society.
